# Epidemiology and Perioperative Mortality of Exploratory Laparotomy in Rural Ghana

**DOI:** 10.5334/aogh.2586

**Published:** 2020-02-25

**Authors:** Brandon S. Hendriksen, Laura Keeney, David Morrell, Xavier Candela, John Oh, Christopher S. Hollenbeak, Temitope E. Arkorful, Richard Ofosu-Akromah, Evans K. Marfo, Forster Amponsah-Manu

**Affiliations:** 1 Department of Surgery, The Pennsylvania State University, College of Medicine, Hershey, PA, US; 2 Department of Health Policy and Administration, The Pennsylvania State University, University Park, PA, US; 3 Department of Surgery, Eastern Regional Hospital, Koforidua, GH

## Abstract

**Background:**

Perioperative mortality rate (POMR) has been identified as an important measure of access to safe surgical and anesthesia care in global surgery. There has been limited study on this measure in rural Ghana. In order to identify areas for future quality improvement efforts, we aimed to assess the epidemiology of exploratory laparotomy and to investigate POMR as a benchmark quality measure.

**Methods:**

Surgical records were reviewed at a regional referral hospital in Eastern Region, Ghana to identify cases of exploratory laparotomy from July 2017 through June 2018. Patient demographics, health information, and outcomes data were collected. Logistic regression was used to identify predictors of perioperative mortality.

**Findings:**

The study included operations for 286 adult and 60 pediatric patients. Only 60% of patients were covered by National Health Insurance (NHI). The overall POMR was 11.5% (12.6% adults; 6.7% pediatric). Sixty percent of mortalities were referrals from outside hospitals and the mortality rate for referrals was 13.5%. Odds of mortality was 13 times greater with perforated peptic ulcer disease (OR = 13.1, p = 0.025) and 12 times greater with trauma (OR = 11.7, p = 0.042) when compared to the most common operation. Female sex (OR = 0.3, p = 0.016) and NHI (OR = 0.4, p = 0.031) were protective variables. Individuals 60 years and older (OR = 3.3, p = 0.016) had higher mortality.

**Conclusion:**

POMR can be an important outcome and quality indicator for rural populations. Interventions aimed at decreasing emergent hernia repair, preventing perforation of peptic ulcer disease, improving rural infrastructure for response to major trauma, and increasing NHI coverage may improve POMR in rural Ghana.

## Introduction

In recent years, there has been an increasing focus on the global burden of surgical diseases [1]. In 2015, the Lancet Commission on Global Surgery showed that the world’s poorest 5 billion people lack access to safe, affordable surgical and anesthesia care [2]. In developing a strategy to meet this need, the perioperative mortality rate (POMR) was identified as one of six core indicators for monitoring universal access to safe surgical care and anesthesia care. As a global indicator, the POMR is defined as the in-hospital mortality due to any cause during surgery over the number of patients undergoing an operation [3]. The POMR can thus be used as a tool to identify procedures that carry the highest mortality rates; identifying the causes of mortality can assist in setting priorities for improving outcomes.

Even before the inception of the six core indicators, overall improvement in surgical services in sub-Saharan Africa was a priority. The Bellagio Essential Surgery Group (BESG) described a need for quantification of surgical outcomes not only on a country level but also between populations with different characteristics, for example, urban and rural [4].

In Ghana, the majority of studies on surgical outcomes and POMR has come from teaching hospitals in Kumasi and Accra—two large hospitals located in urban regions. [5,6,7,8]. The generalizability of this data to hospitals serving more rural Ghana is not clear.

Eastern Regional Hospital (ERH) serves as a surgical referral center located in Koforidua, Ghana, for the 2.5 million people of Eastern Region, Ghana, of which 56% is classified as rural [9]. On average patients travel 98 km to arrive at the facility. Surgical disease is frequently a reason for referral as many district hospitals are not equipped with the facilities or personnel required for major abdominal operations [10]. Therefore, one of the most common operations performed at this facility is emergent exploratory laparotomy. Late disease presentation combined with a frequent lack of pre-operative imaging makes these operations particularly challenging.

The purpose of this study was to quantify surgical outcomes for exploratory laparotomy with a specific focus on POMR for a rural referral center in Ghana. Our aim was to assess the factors associated with POMR in order to identify potential areas for process improvement.

## Methods

### Data sources

Data were obtained retrospectively from the surgical logbook at ERH in Koforidua, Ghana. Included in the data was a medical record number, which was used to obtain patient electronic medical records (EMR). Data from the surgical logbook and the electronic medical records were compiled and corroborated.

Approval for use of the data was given by the institutional review board at The Pennsylvania State University College of Medicine (STUDY00009316) as well as by ERH leadership.

### Population

The study included all patients who underwent an exploratory laparotomy between July 1, 2017 and June 30, 2018 at ERH. Exploratory laparotomy was defined as an open abdominal operation in an emergent or urgent setting. Patients with planned open abdominal operations, such as stoma reversals, were not included in the study. All operations were supervised by a single general surgeon. Pediatric (0–17) and adult (18+) sub-groups were created for analysis. Patients were excluded for discrepancies between the logbook and EMR.

The population was further stratified for a secondary analysis beginning in December of 2017. Patients who were initially seen at an outside hospital, as opposed to patients who initially presented to ERH, were considered as referrals.

### Outcomes

Peri-operative in-hospital mortality was the primary outcome of the study. Secondary outcomes included hospital length of stay (LOS), 30-day readmission, 30-day re-laparotomy, surgical site infection (SSI), and bowel resection.

### Covariates

Analyses controlled for demographic and clinical covariates. Demographic covariates included age, sex (male, female), marital status (single, married, widowed, divorced, unknown), occupation (laborer, professional, student, unemployed, unknown), and insurance (National Health Insurance [NHI], cash). Clinical covariates included hemoglobin levels prior to operation (0–11.9, 12.0–14.9, 15+, unknown), white blood cell (WBC) counts prior to operation (0–3.99, 4–10.99, 11–14.99, 15+, unknown) and post-operative diagnosis (appendicitis, obstruction, perforated peptic ulcer disease, typhoid ileitis, major trauma, intussusception, other). Covariates used in multivariable modeling were limited in order to avoid overfitting the model.

### Statistical analysis

The key aims of the study were, first, determine the most common diagnoses associated with exploratory laparotomy, second, assess the impact of those diagnoses on POMR, third, identify the rates of other short-term perioperative outcomes, and fourth, determine the impact of referrals on the surgical mortality rate at ERH.

Chi squared tests were used to compare outcomes between adult and pediatric patients. Multivariable logistic regression modeling of mortality was performed for all patients. The sub-group analysis of impact of referrals on mortality was assessed with a chi squared test. The software used to perform the statistical analysis was STATA (version 10.1, StataCorp, College Station, TX, USA). Statistical significance was defined by p-value < 0.05.

## Results

The study identified 346 patients (286 adult and 60 pediatric) who underwent exploratory laparotomy between July of 2017 and June of 2018 and met inclusion criteria.

Table 1 shows characteristics of patients undergoing exploratory laparotomy. The average age of the adult patient was 46.5 years (SD 18.2). Males accounted for slightly more than 60% of all patients. Adults were most commonly married and employed as laborers. Of all adults, 59.1% were insured by the National Health Insurance Authority (NHIA) at the time of operation. Conversely, 38.8% had to cover health care costs out-of-pocket. Seventy percent had laboratory evaluation of hemoglobin and WBC levels prior to surgery. Of those patients, 28.3% were anemic (hemoglobin level less than 12.0) and 32.9% had an elevated WBC count (WBC greater than or equal to 11.0).

**Table 1:** Baseline characteristics of patients prior to exploratory laparotomy.

Variable	Adult (n = 286)		Pediatric (n = 60)	

	n	%	n	%

Age (mean years)		46.5		9.4
Sex				
Male	179	62.6%	45	75.0%
Female	107	37.4%	15	25.0%
Marital status				
Single	77	26.9%	56	93.3%
Married	168	58.7%	2	3.3%
Widowed	23	8.0%	0	0.0%
Divorced	11	3.8%	1	1.7%
Unknown	7	2.4%	1	1.7%
Occupation				
Laborer	143	50.0%	0	0.0%
Professional	25	8.7%	0	0.0%
Student	30	10.5%	52	86.7%
Unemployed	21	7.3%	1	1.7%
Unknown	67	23.4%	7	11.7%
Insurance status				
NHIA	169	59.1%	36	60.0%
Cash	111	38.8%	23	38.3%
Unknown	6	2.1%	1	1.7%
Hemoglobin level				
0–11.9	81	28.3%	25	41.7%
12.0–14.9	60	21.0%	19	31.7%
15.0+	57	19.9%	4	6.7%
Unknown	88	30.8%	12	20.0%
White blood cell count				
0–3.99	13	4.5%	0	0.0%
4–10.99	92	32.2%	21	35.0%
11–14.99	52	18.2%	8	13.3%
15.0+	42	14.7%	19	31.7%
Unknown	88	30.8%	12	20.0%

The average age of the pediatric patient was 9.4 years (SD 5.7) and 75% were males. The vast majority were single (93.3%) but there were teenagers who identified as married or divorced (5.0%). Overwhelmingly, this population identified as students and, similar to the adult population, 60.0% were insured at the time of surgery while 38.3% paid for health costs out-of-pocket. 80.0% of patients had laboratory evaluation prior to surgery. Of those, 73.4% were anemic with a hemoglobin of less than 12.0 and 45.0% had a leukocytosis with a WBC greater than or equal to 11.0.

**Figure 1:**
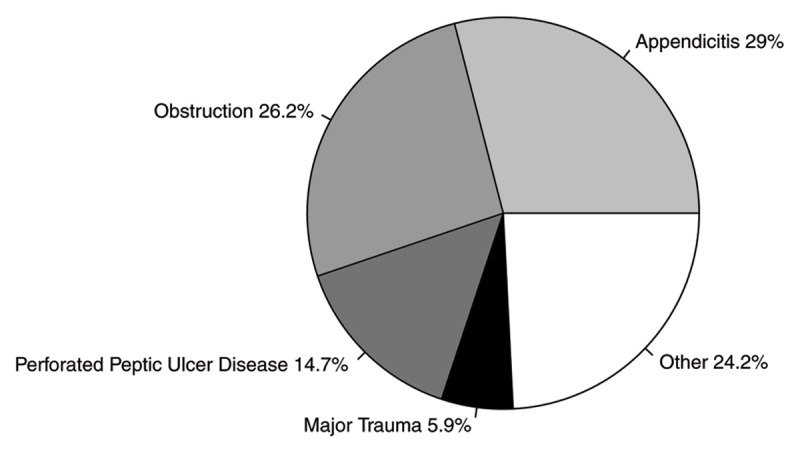
Most common disease processes requiring exploratory laparotomy in the adult population of surgical patients at Eastern Region Hospital, Ghana.

Figure 1 shows diagnoses associated with exploratory laparotomy in the adult population. Appendicitis (29.0%), obstruction (26.2%), perforated peptic ulcer disease (14.7%), and major trauma (5.9%) were the most common indications for an operation. Of note, the cases of appendicitis were complicated by abscess or perforation 59.0% of the time. Causes of obstruction were as follows: hernia (43.0%), adhesions (43.0%), and neoplasia (14.0%). Interestingly, there was only one non-therapeutic exploratory laparotomy performed on a patient with pancreatitis.

**Figure 2:**
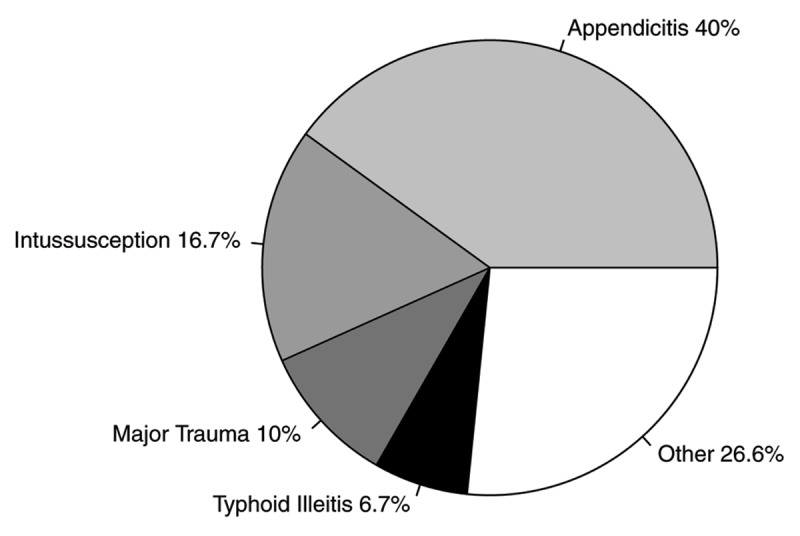
Most common disease processes requiring exploratory laparotomy in the pediatric population of surgical patients at Eastern Region Hospital, Ghana.

Indications for an operation in the pediatric population are depicted in Figure 2. Appendicitis (40%), intussusception (16.7%), major trauma (10%), and typhoid ileitis (6.7%) were most common. There were no negative exploratory laparotomies in the pediatric population.

Rates of perioperative surgical outcomes are listed in Table 2. In the adult population, the mortality rate associated with exploratory laparotomy was 12.6%. The average LOS for a surgical patient was 7.2 days and patients were readmitted within 30 days at a rate of 9.4%. SSIs occurred in 9.8% of adults. In total, 21% of operations included a bowel resection.

**Table 2:** Surgical outcomes following exploratory laparotomy.

Surgical Outcomes	Adult	Pediatric	p-value

	(n = 286)	(n = 60)	

Mortality	12.6%	6.7%	0.192
Length of stay (days)	7.2	6.9	0.758
30-day readmission	9.4%	8.3%	0.788
SSI	9.8%	6.7%	0.448
Bowel resection	21.3%	21.7%	0.954

In the pediatric group, the mortality rate was 6.7% and the LOS averaged 6.9 days. Within 30 days, 8.3% of patients were readmitted to the hospital. SSIs were detected at a rate of 6.7%. About 21.3% of all exploratory laparotomy operations in adults and 21.7% in children included a bowel resection. There were no statistically significant differences between the outcomes of adults and children.

Further stratification of POMR by disease is shown in Table 3. The four most common reasons for exploratory laparotomy for adults and children are shown with respective number of operations and mortality rates. In adults, exploratory laparotomy following major trauma was associated with the highest mortality rate at 17.6%. Intussusception had the highest mortality rate in children at 20.0%.

**Table 3:** Perioperative mortality rates associated with the most common causes of exploratory laparotomy.

Adult			Pediatric		

(Total operations = 286, Overall mortality rate = 12.6%)			(Total operations = 60, Overall mortality rate = 6.7%)		

Disease process	Total operations (n)	Mortality rate (%)	Disease process	Total operations (n)	Mortality rate (%)

1. Appendicitis	83	0.0%	1. Appendicitis	24	4.2%
2. Obstruction	75	14.7%	2. Intussusception	10	20.0%
3. Perforated PUD	42	14.3%	3. Major trauma	6	0.0%
4. Major trauma	17	17.6%	4. Typhoid ileitis	4	0.0%

The results of multivariable modeling of mortality for all patients is shown in Table 4. Female sex (OR = 0.36; p = 0.023) and NHI (OR = 0.41; p = 0.041) were protective against mortality. Unsurprisingly, age 60+ and leukopenia were predictive of mortality (OR = 3.34, p = 0.017 and OR = 5.06, p = 0.031).

**Table 4:** Logistic regression modeling of mortality.

Variable	95% confidence interval			

	OR	Lower	Upper	p-value

Age (years)				
0–17	0.43	0.09	2.00	0.280
18–39	0.45	0.14	1.47	0.184
40–59	Reference			
60+	3.34	1.25	8.94	0.017
Sex				
Male	Reference			
Female	0.36	0.15	0.87	0.023
Insurance status				
NHI	0.41	0.17	0.96	0.041
Cash	Reference			
Diagnosis				
Appendicitis	Reference			
Obstruction	12.23	1.44	104.20	0.022
Perforated PUD	13.09	1.38	124.44	0.025
Typhoid ileitis	18.87	0.94	377.46	0.055
Major trauma	12.14	1.14	129.78	0.039
Intussusception	34.29	2.18	539.35	0.012
Other	24.85	3.03	203.78	0.003
Hemoglobin level				
0–11.9	0.67	0.26	1.69	0.394
12.0–14.9	Reference			
15.0+	0.18	0.04	0.71	0.015
White blood cell count				
0–3.99	5.06	1.16	21.98	0.031
4–10.99	Reference			
11–14.99	1.32	0.45	3.90	0.616
15.0+	2.13	0.67	6.78	0.199

Compared to the reference operation, several diagnoses impacted mortality. Obstruction (OR = 12.23, p = 0.022), perforated peptic ulcer disease (OR = 13.09, p = 0.025), major trauma (OR = 12.14, p = 0.039), and intussusception (OR = 34.29, p = 0.012) significantly increased the odds of death.

**Figure 3:**
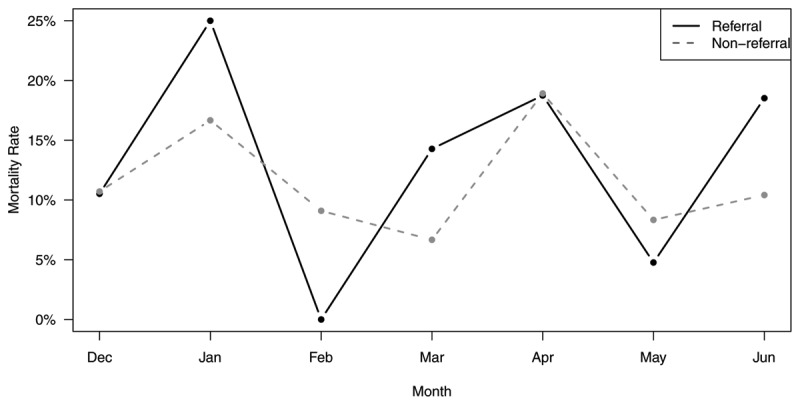
Trends in perioperative mortality rate following exploratory laparotomy stratified by referral status for surgical patients at Eastern Region Hospital, Ghana.

Secondary analysis of the impact of referrals on mortality included 225 patients from the initial analysis (126 referrals and 99 non-referrals). Referred patients accounted for 60% of all mortalities. On average 54% of all patients undergoing exploratory laparotomy were first seen at an outside facility prior to their operation. Trends in mortality rates for referral and non-referral patients are shown in Figure 3. Overall, the mortality rate for referred patients was 13.5% and the mortality rate for non-referred patients was 11.1% These mortality rates were not significantly different (p = 0.591).

## Discussion

At first glance, an overall surgical mortality rate of 12.6% for exploratory laparotomy in the adult population of our study seems high given prior estimates of global POMR of 0.8%–1.5% [11]. However, that study included all operations, across varying degrees of operative urgency, and with extensive variation in surgical disease. More likely, the elevated mortality rate demonstrates the complexity and advanced degree of surgical disease requiring laparotomy at ERH. In fact, in a study using data from 83 low- and middle-income countries (LMIC), the POMR for laparotomy was estimated to be 11.6%, which closely mirrors the mortality rate of laparotomy at ERH [12].

Appendicitis, obstruction, perforated peptic ulcer disease, and major trauma were the most common surgical disease processes in adults. Additionally, intussusception and typhoid ileitis were common in the rural pediatric population of ERH. Similarly, appendicitis, typhoid ileitis, obstruction, and gastroduodenal perforations were reported as the major reasons for emergent laparotomy in a larger facility serving a nearby urban population in Ghana [5]. The notable exception was the significantly higher rates of typhoid ileitis at the urban facility.

Operative intervention for appendicitis was remarkable in that it was the most common reason for surgery in both the adult and pediatric population. While the POMR for these operations was very low, these operations were not without associated mortality in the pediatric population. Likely contributing to this was the associated 59% rate of complicated appendicitis found at the time of surgery. In the near future we aim to investigate the drivers of complicated appendicitis, including delayed presentation.

Overall, the major causes of obstruction identified at ERH were hernia (43%), adhesions (43%), and neoplasia (14%). Interestingly, in the more urban capital of Accra, the rates of obstruction due to hernia have dramatically decreased such that hernias are now the eighth most common reason for obstruction. This has been attributed to an increase in the surgical workforce and a conscious effort to reduce hernia “backlog” in the capital [13]. Unfortunately, the changes in urban Ghana do not yet appear to have reached rural Ghana where emergent and urgent operations for hernias likely increase POMR. While it would be ideal to similarly increase the surgical workforce at ERH, perhaps more realistically, we could aim to better educate the population to have hernias repaired earlier and in an elective fashion. Engaging community health workers could be effective with outreach and education in rural communities [14].

Perforated peptic ulcer disease resulted in one of the highest mortality rates at 14.3%. A comparison rate of 13.6% was reported in a systematic review of the literature of sub-Saharan Africa [15]. More importantly, the high mortality rate was associated with an impressive case load as PUD accounted for nearly 15% of all exploratory laparotomies. The high incidence of perforation is likely multi-factorial. *H. pylori* prevalence ranges between 45%–80% in Ghana [16,17]. The use of herbal remedies and NSAIDs is also thought to contribute to the problem [18]. Going forward, a multi-disciplinary approach to earlier identification and treatment of peptic ulcer disease could greatly impact POMRs and surgical burden.

The POMR for major abdominal trauma requiring operative intervention in our study was approximately four times that reported in nearby urban centers (17.6 % versus 4.4%) [6]. The contributions of limited surgical workforce, distance to the hospital, and limited emergency transportation services are a few factors that need to be considered and investigated for quality improvement. In general, trauma in rural settings of LMICs has been a significant cause of increased morbidity and mortality. However, there is some evidence to suggest that systems-based interventions can lead to decreases in the mortality rates [19,20].

In our pediatric cohort, typhoid ileitis and intussusception were among the most common indications for laparotomy. Typhoid intestinal perforation can be fatal [12,21]. However, recent public health initiatives, improved sanitation, and operative intervention have been credited for an overall decrease in the incidence and mortality associated with typhoid intestinal perforation in LMICs, including Ghana [22,23,24]. Intussusception had the highest mortality rate in our study at 20%. In contrast to high income countries (HICs), radiographic decompression is not frequently available, and thus, children require operative intervention. The estimated mortality rate associated with intussusception for children in LMICs ranges from 6–25% among those who undergo surgery, compared to <1% in HICs [21].

Importantly, patients with NHI had improved POMRs. Odds of perioperative survival was 60% higher in patients who had health insurance. In both adults and children, nearly 40% of patients did not have insurance despite a low annual premium of $5 per person, and with children automatically covered if both parents have insurance. Interestingly, approximately 55% of all Ghanaians were thought to be enrolled with NHI in 2009 [25]. This suggests that either the overall enrollment rate has stagnated or efforts to insure Ghanaians have not reached rural areas. Interventions aimed at improving NHI coverage could have an important impact on POMR in Ghana.

There are a number of limitations in our study. First, more research data points from across the country of Ghana are needed to draw conclusions about differences in urban and rural settings. Next, and perhaps most notably, is the inability to risk adjust the POMR due to lack of retrospectively available patient information. Ideally, factors including, but not limited to, American Society of Anesthesiologist physical status classification, wound classification, functional status, and age should be used [3]. Adjunct therapies, such as anti-secretory therapy in patients with peptic ulcer disease, were also not reliably recorded in the EMR preventing inclusion in this study. Study outcomes related to mortality were limited to inpatient mortality given inability of the EMR to reliably capture 30-day and 90-day mortality rates. Going forward, our aim is to improve documentation of factors necessary for risk adjustment at this institution. Due to the small sample size, the power of the study was also limiting. It is conceivable that factors included in our mortality model could become significant with larger numbers. Additionally, sample size likely impacted our analysis of the influence of referrals on POMR. Finally, extrapolation of our findings to other rural hospitals in Ghana and throughout sub-Saharan Africa should be done with caution as our data points and findings are drawn from the operative experience of a single surgeon.

## Conclusion

POMR has become an important quality indicator within the expanding field of global surgery. Our study shows that the benefits of POMR investigation extend to a hospital and community level and can provide insights into opportunities for future quality improvement initiatives. Additionally, this study demonstrates that collaboration between a large university and a rural hospital can result in meaningful research and quality improvement initiatives.
